# Identification of QTLs for grain yield and other traits in tropical maize under *Striga* infestation

**DOI:** 10.1371/journal.pone.0239205

**Published:** 2020-09-14

**Authors:** Baffour Badu-Apraku, Samuel Adewale, Agre Angelot Paterne, Melaku Gedil, Johnson Toyinbo, Robert Asiedu

**Affiliations:** International Institute of Tropical Agriculture (IITA), Ibadan, Nigeria; University of Helsinki, FINLAND

## Abstract

*Striga* is an important biotic factor limiting maize production in sub-Saharan Africa and can cause yield losses as high as 100%. Marker-assisted selection (MAS) approaches hold a great potential for improving *Striga* resistance but requires identification and use of markers associated with *Striga* resistance for adequate genetic gains from selection. However, there is no report on the discovery of quantitative trait loci (QTL) for resistance to *Striga* in maize under artificial field infestation. In the present study, 198 BC_1_S_1_ families obtained from a cross involving TZEEI 29 (*Striga* resistant inbred line) and TZEEI 23 (*Striga* susceptible inbred line) plus the two parental lines were screened under artificial *Striga-*infested conditions at two *Striga*-endemic locations in Nigeria in 2018, to identify QTL associated with *Striga* resistance indicator traits, including grain yield, ears per plant, *Striga* damage and number of emerged *Striga* plants. Genetic map was constructed using 1,386 DArTseq markers distributed across the 10 maize chromosomes, covering 2076 cM of the total genome with a mean spacing of 0.11 cM between the markers. Using composite interval mapping (CIM), fourteen QTL were identified for key *Striga* resistance/tolerance indicator traits: 3 QTL for grain yield, 4 for ears per plant and 7 for *Striga* damage at 10 weeks after planting (WAP), across environments. Putative candidate genes which encode major transcription factor families WRKY, bHLH, AP2-EREBPs, MYB, and bZIP involved in plant defense signaling were detected for *Striga* resistance/tolerance indicator traits. The QTL detected in the present study would be useful for rapid transfer of *Striga* resistance/tolerance genes into *Striga* susceptible but high yielding maize genotypes using MAS approaches after validation. Further studies on validation of the QTL in different genetic backgrounds and in different environments would help verify their reproducibility and effective use in breeding for *Striga* resistance/tolerance.

## Introduction

In sub-Saharan Africa (SSA), maize (*Zea mays* L.) is the most widely cultivated cereal crop, playing important food and nutritional roles in the livelihoods of over 300 million households in the sub-region [1–3]. The parasitic purple witchweed *Striga hermonthica* (Del.) Benth. is a serious threat to food and nutrition security, adversely affecting the production of economically important cereal crops such as maize, pearl millet and sorghum [4, 5]. In the savannas of West and Central Africa (WCA), infestation of cereal production areas by *Striga* spp is estimated to be 40 million hectares whereas about 70 million ha have moderate levels of infestation [6]. As a result of the significant level of *Striga* infestation in this sub-region, several maize farmers have deserted their farms due to the complexity of managing *Striga-*infested fields and the high yield reduction in such fields. Damage caused by *Striga* parasitism has been reported to be intensified by low soil N and drought, the typical predominant conditions on smallholder farmers’ fields in *Striga*‐prone regions in Africa [7]. The difficulty in controlling the devastating impacts of *Striga* on the growth and yield of cereals may be attributed to its dual mode of action (i) *Striga* plants compete effectively with the host for carbon, nitrogen and inorganic solutes, and (ii) the parasite has a ‘phytotoxic’ effect on the host plant within few days of attachment [4, 8]. A diverse array of control measures being employed to mitigate the effects of *Striga* includes intercropping, crop rotation, use of herbicides, hand pulling, trap and catch crops, high nitrogen fertilization, and planting of *Striga* resistant varieties [3]. Although no single control option is capable of achieving total control of the parasite, the breeding for genotypes that combine resistance and tolerance is widely recognized as the most economically feasible and sustainable strategy for controlling the parasite [9]. As a result, maize breeders at IITA have embarked upon breeding for host plant resistance in the past three decades as a major control option [10, 11] for sustainable control of *Striga* parasitism in maize. In *Striga* research, resistance to *Striga* refers to the ability of the host plant to stimulate the germination of *Striga* seeds but prevent the attachment of the parasites to its roots or kill the attached parasites. Under *Striga* infestation, the resistant genotype supports significantly fewer *Striga* plants and produces greater yield than a susceptible (converse of resistance) genotype [12–14]. In contrast, tolerance to *Striga* (converse of sensitivity) refers to the ability of the host plant to support equal levels of *Striga* infestation as the intolerant or sensitive genotype [9], without associated impairment of growth or grain yield losses [15, 16]. *Striga* resistance and tolerance are highly complementary defense mechanisms. A combination of these mechanisms is the most promising breeding strategy for reducing *Striga* infection and reproduction levels in infested fields, while maintaining yield levels and ensuring food security for the resource-poor farmers [9, 10, 13, 17, 18].

To employ host plant resistance/tolerance for effective and sustainable control of *Striga hermonthica*, several studies have been executed to understand the genetic basis of maize resistance/tolerance to the *Striga* parasitism [11, 19–23]. However, reports of earlier researchers on the mode of gene action of *Striga* resistance in maize have been contradictory, taking into consideration *Striga* resistance/tolerance indicator traits such as grain yield in *Striga* infested environments, severity of host plant damage and number of emerged *Striga* plants on the host. Results of some studies have indicated that resistance/tolerance to *Striga* is polygenically controlled with additive genetic effects more predominant than non-additive effects in regulating the host plant damage severity and grain yield under infestation [11, 19–22]. Contrarily, results of several other studies have revealed the predominance of non-additive gene action in regulating the inheritance of host plant damage, whereas additive gene action was more important in the inheritance of the number of emerged *Striga* plants [10, 21, 23–25]. The implication is that contrasting genes regulate the number of emerged *Striga* plants and host plant damage severity [11, 19]. The findings of far-reaching studies executed by IITA researchers have identified host plant damage severity, emerged *Striga* plants, ears per plant as well as high grain yield in *Striga-*infested environments as key traits for use in the genetic enhancement of maize for *Striga* tolerance/resistance [10]. As criteria for tolerance, host plant *Striga* damage severity was used whereas the number of emerged *Striga* plants indicated the level of resistance. Nevertheless, the genotypic correlation between host damage syndrome rating and emerged *Striga* plants has been reported to be low, indicating that the two traits are under different genetic control [10, 11]. These findings corroborate the hypothesis of Kim [11] and Berner et al [19] who reported that several genes regulate *Striga* emergence and the severity of host plant damage.

During the past few years, the recent advances in molecular marker technologies have facilitated the construction of high-density genetic linkage maps and detection of novel QTL associated with quantitative traits in segregating populations and the characterization of the map positions in the genome of crop plants [26–28]. In maize, studies on QTL identification for complex traits have focused mainly on abiotic stresses such as drought [29–31] and low soil nitrogen [32, 33] and good progress has been made. Also, significant advances have been made in the identification of QTLs in segregating populations under biotic stresses such as the Maize Lethal Necrosis (MLN), Southern corn rust, and Tar Spot Complex (TSC). For example, Gowda et al [34] used GWAS on two diverse maize panels and mapped 24 SNP markers with major and minor effects linked to QTL for resistance to MLN. Wanlayaporn et al [35] used 157 SSR markers to map 15 QTL for partial resistance to southern corn rust, spread across the chromosomes in a mapping population of 69 tropical sweet corn recombinant inbred lines (RILs). However, very limited breakthroughs have been achieved in the identification of QTLs for *Striga* resistance/tolerance in maize.

The employment of marker-assisted selection (MAS) could be a rapid and efficient approach in breeding for resistance/tolerance to *Striga* parasitism. However, the efficiency of MAS is dependent on the identification of closely associated molecular markers or QTLs, which are cost-effective and easy to use. The QTL detected in breeding populations after validation could be useful for crop genetic enhancement through MAS approaches [36, 37]. Thus, identification of QTLs closely linked to *Striga* resistance/tolerance traits in maize is necessary to promote accelerated and efficient transfer of *Striga* resistance/tolerance genes into susceptible maize cultivars. The discovery of *Striga* resistance/tolerance QTL will enhance the understanding of the genetic basis of phenotypic variance and provide new insights for improving maize yields under *Striga* infestation in breeding programs in SSA. However, limited reports have been published on the identification of QTLs for *Striga* resistance/tolerance in maize. For example, Amusan [37] identified two putative loci for resistance to *Striga* on chromosome 6 of maize, using SSR markers and composite interval mapping (CIM) in a late maturing maize F_2_ mapping population. These two QTLs accounted for 55 per cent of the phenotypic variation (PV) with predominance of dominance genetic effects over additive genetic effects in the expression of the two *Striga* resistant QTLs. Similarly, Adewale et al [38] identified significant loci on chromosomes 9 and 10 of maize, closely linked to ZmCCD1 and amt5 genes, respectively which could be related to plant defense mechanisms against *Striga* parasitism. In another study, Badu-Apraku et al. [39] identified twelve QTLs for four *Striga* resistance/tolerance adaptive traits in an F_2:3_ mapping population involving the *Striga* resistant inbred line TZEEI 79 and the *Striga* susceptible inbred TZdEEI 11. The identified QTLs were found to be linked to candidate genes which may be associated with plant defense mechanisms in *Striga* infested environments.

The extra-early (80–85 days to physiological maturity) maturing inbred line TZEEI 29 developed in the IITA-MIP combines *Striga hermonthica* resistance/tolerance with tolerance to low soil N and drought [40]. The inbred line is also an outstanding tester characterized by significant and positive general combining ability (GCA) effects for grain yield under *Striga* infestation, low N and drought. Additionally, the inbred possesses negative and significant GCA effects for *Striga* damage and number of emerged *Striga* plants under *Striga*-infested environments. Furthermore, the inbred has negative and significant GCA effects for stay-green characteristic under drought and low N. Finally, the inbred is the source of several outstanding, *Striga*-resistant commercial open-pollinated varieties and hybrids released in several countries in WCA and is serving as an outstanding inbred tester. Inbred TZEEI 29 was therefore considered a candidate inbred for QTL identification for MAS. The objectives of this study were to (i) identify QTLs associated with *Striga* resistance/tolerance using inbred TZEEI 29 and the extra-early *Striga* susceptible inbred, TZEEI 23, and (ii) identify putative candidate genes underlying the identified QTLs.

## Materials and methods

### Plant materials

The two extra-early white maize parental inbreds used in the present study varied significantly in their responses under artificial *Striga* infestation. The inbred line TZEEI 29 is *Striga* resistant/tolerant while TZEEI 23 is highly susceptible to *Striga* parasitism. The two parental lines were selected from the results of previous studies conducted under *Striga*-infested environments [40]. The *Striga*-susceptible extra-early maize inbred line—TZEEI 23 (TZEE-W SR BC_5_ x 1368 STR S_7_ Inb. 80) and *Striga* resistant/tolerant inbred line—TZEEI 29 (TZEE-W SR BC_5_ x 1368 STR S_7_ Inb. 27) used in the present study were derived from a cross between TZEE-W SR BC_5_, a *Striga* susceptible extra-early variety and the *Striga*-tolerant late maturing IITA inbred line TZi 3 (1368 STR) which were crossed to initiate the development of an extra-early maturing, *Striga* resistant population to serve as a source of *Striga* and drought tolerant inbred lines for hybrid development. The cross (TZEE-W SR BC_5_ x 1368 STR) F_1_ was backcrossed twice to TZEE-W SR BC_5_ to recover extra-earliness. This resulted in the development of the extra-early population, [(TZEE-W SR BC_5_ x 1368 STR) x TZEE-W SR BC_5_] which was taken through seven cycles of inbreeding and selection under artificial *Striga* infestation and induced moisture stress to obtain several *Striga* resistant/tolerant and drought tolerant inbred lines including TZEEI 29 and the *Striga* susceptible inbred line TZEEI 23. Thus, a cross between these genetically diverse maize genotypes resulted in the highly *Striga* resistant/tolerant parental line TZEEI 29 and highly susceptible line TZEEI 23. For the development of theBC_1_S_1_ mapping population, crosses were made between TZEEI 29 and TZEEI 23 designated as P_1_ and P_2_ respectively, to obtain 1200 F_1_ progenies. The F_1_ hybrids along with the parental lines were planted and leaf samples were collected at 3 weeks after planting for quality control (i.e. verification of true-to-type F_1_ progenies). The F_1_ progenies were screened using two SSR primers (phi072 and umc1568) which were found to be polymorphic across the two parents [41]. Based on the results of the quality control, 650 true-to-type F_1_ hybrids (S1 Table) were backcrossed to the susceptible inbred, TZEEI 23 to obtain 650 BC_1_F_1_ genotypes. After eliminating undesirable ears due to ear rot, 260 BC_1_F_1_ individuals were genotyped and selfed to obtain BC_1_S_1_ families, for phenotyping in replicated trials at two locations. The 260 BC_1_F_1_ genotypes were further confirmed as true-to-type by employing the pedigree verification tool in Flapjack software [42], using SNP marker data (S1 Fig, S2 Table). Finally, 198 BC_1_S_1_ families were selected based on the availability of both genotypic and phenotypic data for the linkage map construction and QTL analysis.

### Field evaluations

The one hundred and ninety eight BC_1_S_1_ families (selfed BC_1_F_1_ individual plants) selected for the study were evaluated along with the two parental lines under artificial *S*. *hermonthica* infestation at Mokwa (9^0^18′N, 5^0^4′E, 210m a.s.l, 1100 mm yearly rainfall) and Abuja (9^0^ 16′N, 7^0^ 20′E, 445m a.s.l, and 1500 mm yearly rainfall) in the Southern Guinea savanna of Nigeria in 2018. The experimental field at Mokwa has a luvisol soil type while that at Abuja has ferric luvisol soil type [43]. A 10 × 20 lattice design with two replications was used for the evaluation of the 200 genotypes at the two locations. Each experimental unit comprised 3 m long single row plots with an inter-row spacing of 0.75 m and within-row spacing of 0.4 m. The fields for artificial *Striga* infestation at Mokwa and Abuja were treated with ethylene gas at 2 wk before planting to induce suicidal germination of *Striga* seeds present in the soil. The S. *hermonthica* seeds used for the trials conducted were collected in the previous year from farmer’s sorghum fields around the test locations, Abuja and Mokwa. The artificial *Striga* infestation was carried out as proposed by the IITA Maize Program [10]. About a week before inoculation, the *Striga* seeds were carefully mixed with finely sieved sand at the ratio 1:99 by weight to ensure rapid and uniform infestation. A standard scoop calibrated to deliver about 5000 germinable *Striga* seeds per hill was used for the artificial infestation. Three maize seeds were placed into the holes infested with the sand-mixed *S*. *hermonthica* seeds. The seedlings were later thinned to two plants per stand at two weeks after emergence to obtain a target population density of 66,666 plants/ha. Fertilizer application on the maize plots was delayed till about 30 days after planting, in order to subject the maize plants to stress, a condition that was expected to enhance strigolactone production, thus ensuring enhanced germination of *Striga* seeds and attachment of *Striga* plants to the roots of host plants. At this stage of plant growth, 20–30 kg N/ha, 30 kg each of P and K were applied as NPK 15-15-15 depending on the fertility status of the soil. The reduced rate of fertilizer application was important because *Striga* emergence decreases at high N rate [10]. Data were recorded on the number of emerged *Striga* plants while host plant damage syndrome rating was recorded on a scale of 1–9 where 1 = normal plant growth, no visible symptoms, and 9 = complete scorching of the leaves, resulting in premature death or collapse of host plant with no ear formation [10]. In addition, the number of ears per plant (EPP) was estimated by dividing the total number of ears harvested per plot by the number of plants per plot. Grain yield (kg ha^− 1^) was computed from field weight of ears per plot. A shelling percentage of 80 was adopted and the moisture content was adjusted to 15%. Moisture content at harvest was recorded for representative shelled kernels from each plot using a moisture meter.

### Statistical analysis of phenotypic data

The data recorded on the number of emerged *Striga* plants as well as *Striga* damage severity score were subjected to natural logarithm transformation before analysis of variance (ANOVA). The means of data collected on measured traits per plot were subjected to ANOVA following the Bartlett’s test for homogeneity of variances [44]. The ANOVA was first carried out for each environment. Thereafter, combined ANOVA across environments (locations) was conducted with PROC GLM in SAS using a random statement with the TEST option [45]. Phenotypic and genotypic correlation coefficients were calculated among the traits, using the adjusted means of the BC_1_S_1_ families. The replications and blocks within replications were considered as random and the BC_1_S_1_ families as fixed effects. Broad sense heritability of the traits (ĥ^2^) across environments was estimated on a family-mean basis as described by Holland *et al*. [46].

### Genotyping

Young leaves from single plants of the 198 BC_1_F_1_individuals as well as bulked leaf samples from each of the two parents were collected, freeze-dried using liquid nitrogen and then used for DNA extraction. Genomic DNA was extracted using the DArTseq protocol (www.diversityarrays.com/files/DArT_DNA_isolation.pdf). The quality of the extracted DNA (concentration and purity) was assessed on agarose gel (2%w/v) and analyzed on the ND-1000 spectrophotometer platform (NanoDrop, Wilmington, DE, USA).

Whole-genome genotyping for the 198 BC_1_F_1_ individuals plus the two parents was carried out using DArTseq technology [47, 48]. Genome complexity reduction which involved the use of a combination of two restriction enzymes (*Pst*I-*Mse*I) was used to create a genome representation of the analyzed samples. All fragments generated were amplified and sequenced to identify the single nucleotide polymorphisms (SNPs) using a proprietary analytical pipeline developed by DArT P/L. After a strict quality control process, which included parameters such as call rate, data reproducibility (~20% of samples replicated), and rate of monomorphism to eliminate monomorphic markers, a number of 10,660 SNPs were extracted from the evaluated germplasm.

### Construction of genetic linkage map and QTL mapping

The 10,660 SNPs were filtered for unmapped and duplicate markers, and polymorphic markers were selected. A chi-square goodness-of-fit test was performed to eliminate loci with significant deviation (*P-*value ≤ 0.01) from the expected Mendelian ratio for a backcross (1:1) [49]. After quality filtering, a total of 1,386 markers distributed across the 10 chromosomes were identified as good SNP markers for genetic map construction.

Genetic linkage (GL) map was constructed, and recombination fractions and logarithm of the odds (LOD) scores were displayed using the R/qtl package (http://www.rqtl.org). The genetic distances were estimated using the “est.map” function with “kosambi” distance from the R/qtl package [50, 51]. SNP markers across the chromosomes were ordered with pairwise linkage analysis using the “est.rf” function. Linkage groups (LGs) were assigned using the genomic positions of SNP markers determined during the SNP calling. Quantitative trait locus mapping was carried out using the R/qtl software package with composite interval mapping (CIM) method [50, 52]. Each QTL interval was tested for significance using the likelihood-ratio (LOD score). The significance threshold of the LOD score (P = 0.05) was estimated using 1,000 permutations [53]. The additive (Add) and dominance (Dom) effects and the proportion of phenotypic variation explained (PVE%) by each QTL were estimated using the “fitqtl” function of R version 3.3.4. The sign of the additive effect of each QTL was used to identify the origin of the favorable alleles [54]. For *Striga* damage, negative additive effect was considered as the source of favourable allele for the resistant/tolerant parent whereas for grain yield and number of ears per plant positive additive effect was considered as the source of favourable alleles for the resistant/tolerant parent. Identified QTLs were named based on conventional method described by Bo et al [55]. For example, *qgy3* represented the QTL identified for grain yield on chromosome 3.

### Identification of candidate genes

In order to identify putative genes for *Striga* resistance/tolerance, the physical positions of the identified QTLs were mapped against the MaizeGDB database version 4 (RefGen_v4) using the reference genome of the maize B73 inbred line. Candidate genes were mined within the flanking sequences of QTL detected for the *Striga* resistance/tolerance indicator traits.

## Results

### Phenotypic evaluation of the BC_1_S_1_ mapping population

**Fig 1:**
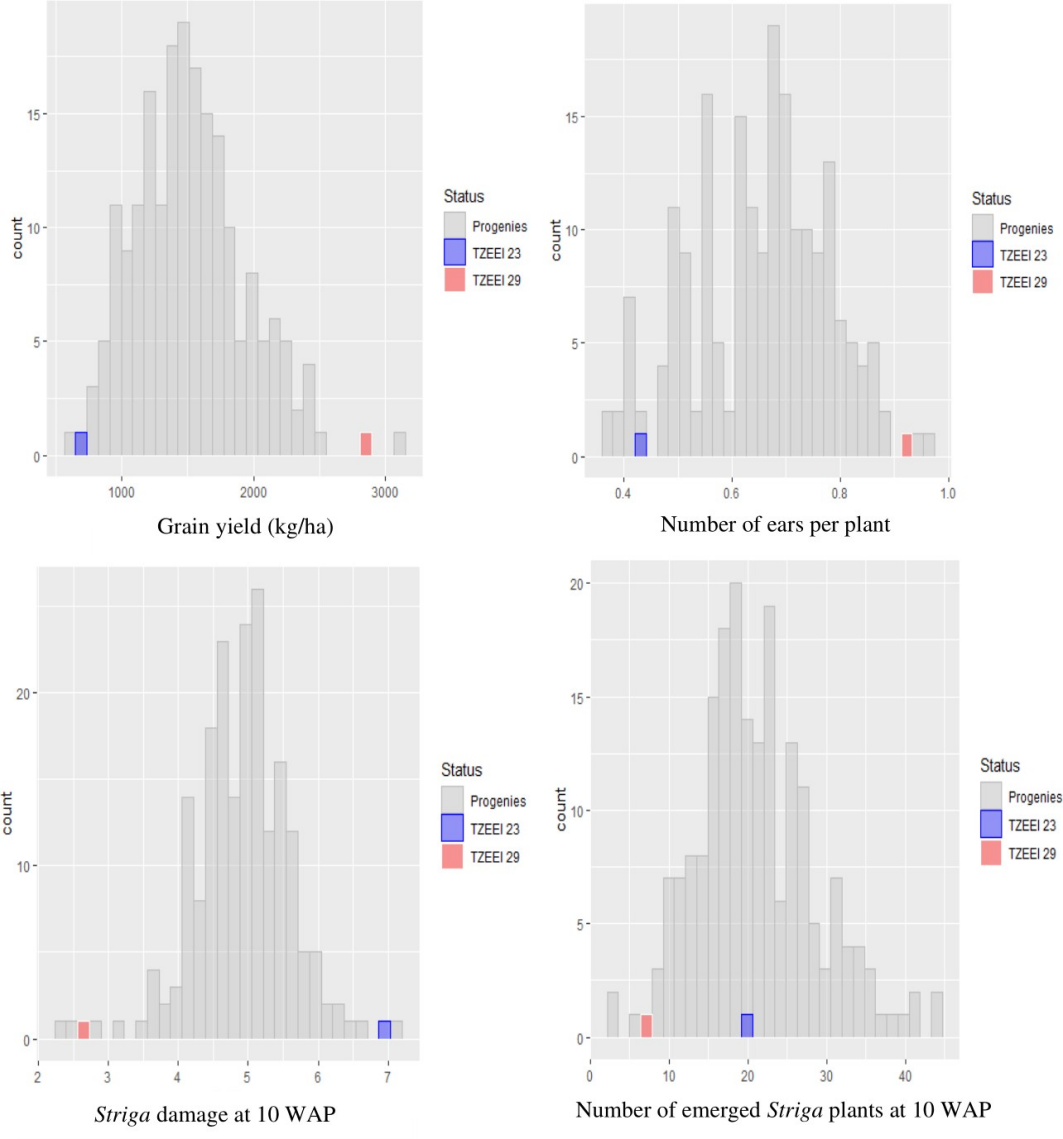
Frequency distribution of grain yield, ears per plant, number of emerged *Striga* plants and *Striga* damage in BC_1_S_1_ population across artificial *Striga* infested environments, in 2018.

Under artificial *Striga* infestation, the target traits recorded in the BC_1_S_1_ population displayed a continuous distribution, varying from highly resistant/tolerant to completely susceptible (Fig 1). In the combined ANOVA, significant mean squares of genotypes and environments were observed for all traits and significant genotype x environment interaction (GEI) mean squares only for the number of emerged *Striga* plants at 10 WAP (Table 1). Similarly, significant mean squares for the genotypes were observed for all traits assayed under *Striga* infestation at Mokwa and Abuja except for the number of emerged *Striga* plants at 10 WAP at Abuja. Across environments, the broad sense heritability of the traits varied from 8% for number of emerged *Striga* plants at 10 WAP to 48% for *Striga* damage at 10 WAP. Moderate heritability estimates (>41%) were recorded for grain yield, ears per plant and *Striga* damage at 10 WAP across research environments (Table 1). Furthermore, significant positive genotypic correlations were recorded between grain yield and ears per plant (r_g_ = 0.84**) while significant negative genotypic correlations were observed between grain yield and *Striga* damage at 10 WAP (r_g =_ -0.96**) as well as ears per plant and *Striga* damage at 10 WAP (r_g_ = -0.77**) (Table 2). Similarly, significant positive phenotypic correlations were obtained between grain yield and ears per plant (r_p_ = 0.70**) while significant negative correlations were observed between grain yield and *Striga* damage at 10 WAP (r_p_ = -0.70**). Additionally, ears per plant displayed significant negative correlations with *Striga* damage at 10 WAP (r_p_ = -0.63**).

**Table 1:** Mean squares of BC_1_S_1_ mapping population evaluated under artificial *Striga* infestation at Abuja, Mokwa and across locations during the 2018 growing season.

Source	Df	Yield, kg/ha	Ears per plant	*Striga* damage rating at 10 WAP	Emerged *Striga* plants at 10 WAP
			Abuja		
Block (Rep)	38	1891936.1**	0.05**	1.65**	1.46**
Rep	1	287496.0	0.07	1.44	19.38**
Genotype	199	662677.4**	0.04**	1.31**	0.60
Error	161	412841.8	0.03	0.68	0.57
Heritability		0.42	0.33	0.51	0.08
			Mokwa		
Block (Rep)	38	1807879.7**	0.18**	3.46**	1.10**
Rep	1	6318217.2**	0.59**	1.21	63.78**
Genotype	199	414118.7**	0.05**	1.03**	0.68**
Error	161	261847.9	0.03	0.73	0.39
Heritability		0.39	0.25	0.34	0.44
			Across		
Env	1	188857529.8*	1.91**	67.28**	10.30**
Block (Rep*Env)	76	176264.8**	0.10**	2.33**	1.21**
Rep (Env)	2	3302856.6**	0.33**	1.33	41.58**
Genotype	199	701684.1**	0.06**	1.56**	0.66**
Genotype*Env	199	415402.2	0.03	0.81	0.63*
Error	322	366577.6	0.04	0.76	0.49
Heritability		0.41	0.44	0.48	0.08

**Table 2:** Genotypic (lower diagonal) and phenotypic (upper diagonal) correlation coefficients between agronomic traits evaluated under artificial *Striga* infestation across Mokwa and Abuja, Nigeria in 2018.

	Grain yield	Ears per plant	*Striga* damage rating at 10 weeks after planting	Emerged *Striga* plants at 10 weeks after planting
Grain yield	-	0.70**	-0.70**	-0.02
Ears per plant	0.84**	-	-0.63**	0.05
*Striga* damage rating at 10 WAP	-0.96**	-0.77**	-	-0.04
Emerged *Striga* plants at 10 WAP	-0.05	-0.61	0.56	-

### Construction of genetic linkage map and QTL mapping

**Fig 2:**
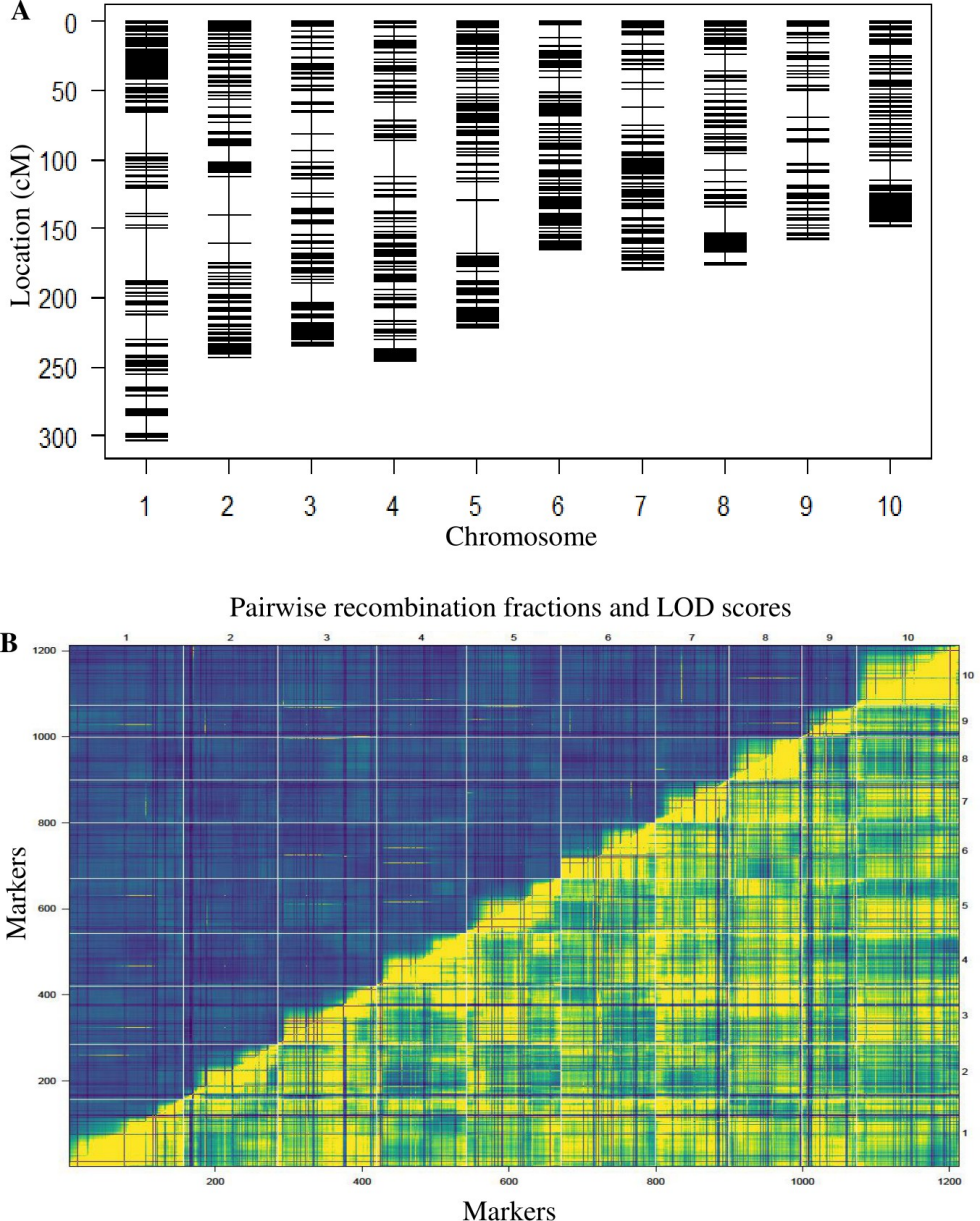
Genetic map derived from the BC_1_S_1_ population. (A) The genetic map with each marker is represented with a horizontal line; (B) Pairwise recombination fractions and LOD scores for all pairs of markers displayed in the upper left and lower right triangle, respectively.

Identification of molecular markers uncovering enough polymorphism among parental lines is crucial for the construction of genetic linkage map. In the present study, the 198 BC_1_F_1_ families plus the two parental inbred lines were genotyped using 10,660 DArTseq derived SNP markers. After quality filtering of the 10,660 SNPs for polymorphic markers, unmapped markers and markers with significant segregation distortions were eliminated, and the resulting 1,386 SNPs were used for the construction of a genetic linkage map (Fig 2, S2 Fig) in the BC_1_S_1_ mapping population. The 1,386 SNP markers were mapped to 10 linkage groups covering 2076 cM of the maize genome. The average marker interval was 0.11 cM. The number of markers mapped per linkage group varied from 86 to 183 while the differences in the lengths of the linkage groups ranged from 148.52 cM to 303.84 cM (Table 3).

**Table 3:** Summary of the genetic linkage map constructed from BC_1_S_1_ population derived from a cross between TZEEI 29 and TZEEI 23 using 1,386 markers.

Chromosome	Marker number	Linkage group length (cM)	Average marker interval (cM)
1	183	303.84	1.67
2	139	243.16	1.76
3	155	234.35	1.52
4	137	245.09	1.8
5	142	221.79	1.57
6	147	165.55	1.13
7	111	179.44	1.63
8	116	176.24	1.53
9	86	157.74	1.86
10	170	148.52	0.88
Total	1386	2075.72	-

QTL analysis of the BC_1_S_1_ mapping population revealed significant QTLs for *Striga* resistance/tolerance indicator traits (that is grain yield, ears per plant, *Striga* damage) under *Striga* infestation, in each and across test environments. The QTLs were declared significant at the 5% level of probability threshold based on 1,000 permutations. QTLs associated with grain yield, *Striga* damage and number of ears per plant under *Striga* infestation and their chromosomal positions, peak and flanking markers, logarithm of odds (LOD), additive effects and the proportion of phenotypic variance explained by each QTL are summarized in Table 4, while the profiles of the LOD of the QTLs across the two environments are shown in S3 Fig. Fourteen QTLs were identified for the *Striga* resistance/tolerance indicator traits in the BC_1_S_1_ mapping population with the phenotypic variance explained (PVE) ranging from 3.0 to 18.5%, across environments.

**Table 4:** Detection of QTL associated with *Striga* resistance/tolerance in the BC_1_S_1_ population derived from TZEEI 29 × TZEEI 23 under artificial *Striga* infestation.

Trait	Location	QTL	Chr	Position (cM)	Left marker	Right marker	LOD	PVE (%)	Add	Dom
Yield	Across	*qgy-3*	3	168.1	S3_167753408	S3_168260561	7.29	18.5	106.30	-76.20
		*qgy-7*.*1*	7	63.5	S7_50192369	S7_76137062	6.01	9.9	-31.60	31.70
		*qgy-8*	8	167.9	S8_167235689	S8_176646370	6.06	12.4	6.10	-5.90
	Abuja	*qgy-3*	3	168.1	S3_167753408	S3_169437737	9.52	16.7	75.10	-74.80
		*qgy-5*	5	197.9	S5_197533513	S5_198713377	7.67	4.2	0.15	-0.27
		*qgy-7*.*1*	7	63.5	S7_50192369	S7_76137062	8.45	12.3	-20.40	20.60
		*qgy-8*	8	167.9	S8_167235689	S8_176646370	7.33	12.9	26.50	-26.50
	Mokwa	*qgy-7*.*2*	7	165.3	S7_161517292	S7_165720114	4.05	8.1	-14.20	12.52
Ears per plant	Across	*qepp-1*	1	152.0	S1_152035554	S1_152647928	5.17	3.0	4.40	-5.20
		*qepp-6*	6	152.1	S6_151829970	S6_152838752	3.92	10.8	-14.20	11.30
		*qepp-7*	7	120.9	S7_118687453	S7_122993327	6.33	7.5	0.04	-0.02
		*qepp-8*	8	88.3	S8_86262321	S8_88276242	4.85	9.4	0.02	0.07
	Abuja	*qepp-1*	1	152.0	S1_150289942	S1_152647928	4.11	5.0	2.60	1.62
		*qepp-7*	7	120.9	S7_118687453	S7_122993327	6.74	13.5	9.05	-8.10
		*qepp-10*	10	139.2	S10_138974863	S10_140174620	4.10	8.8	-0.21	0.27
	Mokwa	*qepp-7*	7	120.9	S7_118687453	S7_122993327	2.97	5.6	-0.01	0.07
		*qepp-8*	8	88.3	S8_85392815	S8_88819092	3.05	4.6	0.05	0.03
*Striga* damage	Across	*qsd-1*	1	285.9	S1_283086643	S1_286796894	5.29	7.7	-0.38	0.98
		*qsd-3*	3	144.7	S3_139083866	S3_145234512	7.51	15.7	-0.06	0.06
		*qsd-6*	6	152.1	S6_151829970	S6_153165363	5.92	13.6	0.03	-0.03
		*qsd-7*	7	130.4	S7_128557561	S7_133378830	8.33	16.4	0.04	-0.04
		*qsd-8*	8	167.9	S8_167235689	S8_176646370	6.86	13.8	8.47	-8.04
		*qsd-9*	9	150.6	S9_150294105	S9_154490555	4.81	9.9	-0.03	0.16
		*qsd-10*	10	138.5	S10_137392359	S10_139667199	5.70	11.5	-3.59	3.59
	Abuja	*qsd-1*	1	285.9	S1_283649322	S1_287919983	6.25	8.4	-0.44	0.08
		*qsd-3*	3	144.7	S3_138825271	S3_145234512	7.16	14.7	0.05	-0.05
		*qsd-7*	7	130.4	S7_128557561	S7_133927224	9.82	19.5	0.05	-0.05
		*qsd-8*	8	167.9	S8_166781475	S8_176646370	6.56	13.3	0.21	-0.21
		*qsd-10*	10	138.5	S10_138884851	S10_140174620	5.94	11.2	-0.06	0.06
	Mokwa	*qsd-3*	3	144.7	S3_139083866	S3_145234512	2.97	5.2	-0.93	0.59
		*qsd-6*	6	152.1	S6_150865187	S6_154341415	2.68	3.2	0.40	-0.91
		*qsd-10*	10	138.5	S10_137392359	S10_ 140375515	2.65	4.6	-0.27	0.03

Three QTLs positioned on chromosomes 3, 7 and 8 were discovered for grain yield across environments, accounting for 9.9 to 18.5% phenotypic variance. The largest effect QTL *qgy-3* for grain yield displayed a LOD of 7.3 and explained 18.5% phenotypic variation. This QTL was located between markers S3_167753408—S3_168260561 with an interval of 0.51cM. Similarly, four QTLs with PVE ranging from 3.0 to 10.8% were identified for ears per plant. Of these QTL, the largest effect QTL for ears per plant, *qepp-6* recorded the highest PVE of 10.8% on chromosome 6 (S6_151829970—S6_152838752) with LOD value of 3.9 and marker interval of 1.0. Furthermore, the QTL analysis identified a total of seven QTL for *Striga* damage across environments. The PVE of a single QTL ranged from 7.7 to 16.4%. The QTL *qsd-8* displayed the largest effect and PVE of 13.8%. It was located on chromosome 8 (S8_166781475—S8_176646370) with a LOD of 6.86. The QTL *qsd-3* and *qsd-10* were also identified in the individual environments under *Striga* infestation. The favourable alleles for QTL *qgy-3*, and *qgy-*8 detected for grain yield, *qsd-1*, *qsd-3*, *qsd-9*, and *qsd-10* for *Striga* damage, as well as *qepp-1*, *qepp-7* and *qepp-8* for EPP identified across environments were contributed by the inbred TZEEI 29 while the favourable allele from QTL *qgy-7*.*1* for grain yield, *qepp-6* for EPP as well as *qsd-6*, *qsd-7* and *qsd-8* for *Striga* damage were contributed by the inbred TZEEI 23 [54] (Table 4). It is striking that no LOD peaks were recorded above the significant thresholds for number of emerged *Striga* plants.

It is interesting that some of the QTLs identified for different traits were in the same region of a chromosome. QTL *qgy-8* detected for grain yield was co-localized with *qsd-8* identified for *Striga* damage at 10 WAP. The QTL *qgy-8* and *qsd-8* had the same peak positions and were flanked by the same markers S8_167766750—S8_167850168. Additionally, *qepp-6* and *qsd-6* identified for grain yield and EPP were co-localized at the same position 152.1cM (Table 4).

### Identification of putative candidate genes

The genomic regions of the significant SNPs were examined to identify the protein-coding genes within the confidence interval of the identified QTLs using the SNP data obtained from the maize genetic database (http://www.maizegdb.org/). The QTL analysis led to the identification of 154 candidate genes associated with *Striga* resistance/tolerance traits (S3 Table). Of the 154 candidate genes, 30 were found to have functions related to plant defense responses (Table 5).

**Table 5:** List of potential candidate genes or proteins associated with the identified QTL for key *Striga* resistance indicator traits under artificial *Striga*-infested environments.

Trait	QTL	LG: start-end position*	Gene ID	Sequence description
Grain yield	*qgy-3*	3:167753408–168260561	GRMZM2G085113	te1—terminal ear1
	*qgy-7*.*1*	7:50192369–76137062	GRMZM2G050550	myb153—sucrose responsive element binding protein
			GRMZM2G123119	ereb177—AP2-EREBP-transcription factor superfamily protein
Ears per plant	*qepp-7*	7:118687453–122993327	GRMZM2G027563	bhlh87—bHLH-transcription factor 87
			GRMZM2G125653	wrky53—WRKY-transcription factor 53
			GRMZM2G169149	wrky104—WRKY transcription factor 104
			GRMZM2G141219	ereb143—AP2-EREBP-transcription factor 143
			GRMZM2G117244	myb140—MYB-transcription factor 140
*Striga* damage	*qsd-1*	1:152035554–152647928	GRMZM2G143204	wrky30—WRKY-transcription factor 30
			GRMZM2G028151	ereb184—AP2-EREBP-transcription factor 184
	*qsd-3*	3: 139083866–145234512	GRMZM5G803355	myb51—MYB-transcription factor 51
			GRMZM2G041415	myb8—myb transcription factor8
			GRMZM2G146020	bzip8—bZIP-transcription factor 8
			GRMZM2G079290	psk2—phytosulfokine2
	*qsd-7*	7:128557561–133378830	GRMZM2G045431	bhlh150—bHLH-transcription factor 150
			GRMZM2G139535	hsftf21—Heat shock factor protein 4
			GRMZM2G082586	bhlh105—bHLH-transcription factor 105
	*qsd-9*	9:150294105–154490555	GRMZM2G113060	ereb71—AP2-EREBP-transcription factor 71
			AC149829.2_FG004	bhlh97—bHLH-transcription factor 97
	*qsd-10*	10:137392359–139667199	GRMZM2G301485	hsftf20—HSF-transcription factor 20
Grain yield *Striga* damage	*qgy-8*, *qsd-8*	8:167235689–176646370	GRMZM2G030762	bhlh55—putative DNA-binding domain superfamily protein
			GRMZM2G065971	mgt8—magnesium transporter8
			GRMZM2G087875	cyp26—putative cytochrome P450 superfamily protein
			GRMZM2G056986	MYB-type transcription factor 79
			GRMZM2G038013	hcf60—high chlorophyll fluorescence60
			GRMZM2G169316	MYB transcription factor
			GRMZM2G310431	hsp1—heat shock protein
Ears per plant, *Striga* damage	*qepp-6*, *qsd-6*	6:151829970–153165363	GRMZM2G180328	nactf20—NAC-transcription factor 20
			GRMZM2G456568	nactf112—NAC-transcription factor 112
			GRMZM2G002128	mybr3—MYB-related-transcription factor 3

## Discussion

Marker-assisted selection is an important tool for precision plant breeding as it allows for indirect selection of a trait of interest based on markers linked to such traits. Identification of genomic regions linked to *Striga* resistance/tolerance in maize would speed up the development of *Striga* resistant germplasm by improving the efficiency of deliberate introgression of novel *Striga* resistant/tolerant genes from diverse germplasm sources into high yielding but *Striga* susceptible genotypes [56]. The *Striga* adaptive traits assayed in the present study showed a normal frequency distribution indicating that the BC_1_S_1_ mapping population was a suitable source for QTL mapping. Significant mean squares of the genotypes under each and across environments revealed the presence of substantial genetic variability for resistance/tolerance to *Striga* among the genotypes which could be attributed to the differences in their genetic backgrounds. Differential responses of maize genotypes under *Striga* infestation have been reported by earlier researchers [57, 58]. The significant environmental variations observed for grain yield and other agronomic traits indicated that the research environments were distinct and provided unique information on the individual families of the mapping population. Genotype x environment interaction was not significant for grain yield, ears per plant and *Striga* damage at 10 WAP but was significant for emerged *Striga* plants at 10 WAP. This implied that most individual families performed similarly in the different research environments [3, 33]. Information on heritability is useful for making decisions on the number of years, locations, and replicates required for testing of genotypes and the breeding method that could be employed to improve the traits of interest. It also allows prediction of genetic gain that could be made from selection.

The moderate heritability estimates observed for most traits under each and across environments underscored the consistency in the expression of most traits under the different environmental conditions. The heritability estimates obtained in our study are greater than those reported by Badu-Apraku et al [3] at the most advanced cycle of selection, C_3_ of the population improved through genomic selection and evaluated under *Striga* infestation. Similarly, the heritability values obtained for grain yield and number of ears per plant were greater than those reported by Ribeiro et al [33], under low N conditions.

Significant positive genotypic and phenotypic correlations were recorded between ears per plant and grain yield whereas host plant *Striga* damage displayed significant negative genotypic and phenotypic correlations with grain yield and the number of ears per plant, such that the lower the host plant *Striga* damage, the higher was the grain yield under *Striga* infestation. However, the genotypic and phenotypic correlations between *Striga* damage and the number of emerged *Striga* plants were not significant, suggesting that the two traits are under different genetic control. Similar findings were reported by Badu-Apraku et al [21].

High-resolution linkage maps are needed for precise identification of QTLs linked with target traits. Genetic distances between markers on such linkage maps rely on chromosome recombination in large populations which is accurately phenotyped [59]. In the present study, a linkage map was constructed corresponding to the 10 chromosomes of the maize genome using 1,386 markers, spanning 2076 cM in length. The length of the linkage map constructed in this study was shorter than those reported by Ramekar et al [26], Wang et al [28], and Ertiro et al [60] but longer than those reported by Samayoa et al [61], Zhao et al [62] and Badu-Apraku et al [39]. The differences between our findings and those of earlier researchers could be attributed to the number of markers used and the type and size of the mapping populations.

A primary aim of this study was to identify QTLs linked to *Striga* resistance/tolerance indicator traits using the extra-early maturing white mapping population derived from the *Striga* resistant inbred line, TZEEI 29 and the extra-early *Striga* susceptible inbred line, TZEEI 23 under artificial *Striga* infestation. So far, there are limited reports on the identification of QTLs for *Striga* resistance/tolerance in maize. However, several studies have identified QTL for grain yield and other measured traits in maize under drought [29–31], low soil N conditions [32, 33] and MLN infection [34]. Identification of QTLs for *Striga hermonthica* resistance/tolerance followed by validation of closely linked markers would speed up the breeding process and result in significant gains in selecting for *Striga* resistance/tolerance and grain yield. The QTL analysis identified 14 QTL for grain yield, number of ears per plant and *Striga* damage at 10 WAP under artificial *Striga* infested environments. The co-localization of *qgy-8* for grain yield and *qsd-8* for *Striga* damage at 10 WAP on chromosome 8 as well as *qepp-6* for ears per plant and *qsd-6* for *Striga* damage at 10 WAP on chromosome 6 supported the strong phenotypic and genotypic correlations between these traits. The mapping of grain yield and *Striga* damage as well as ears per plant and *Striga* damage in the same regions indicated that this regions of the chromosome could be a hotspot for genetic improvement of the *Striga* resistance/tolerance indicator traits and that transfer of this regions into maize genotypes through MAS would fast-track the development of maize varieties with improved grain yield in *Striga*-infested environments. Seven of the fourteen QTLs identified across environments had their marker intervals greater than 4cM from their linked markers, implying that it is necessary to further fine map these chromosomal regions to reduce the marker intervals [33]. The QTL *qsd-3* and *qsd-9* identified in the present study were found to be in the same region with those reported by Adewale et al [38] on chromosomes 3 and 9, respectively. In contrast, QTL identified in the present study for *Striga* resistance/tolerance traits are different from those earlier reported by Badu-Apraku et al [39] in an extra-early maturing yellow F_2:3_ mapping population. This finding is not surprising because the sources of resistance of the extra-early white inbred lines used for the development of the extra-early white mapping population were the same as those used for the development of the early white inbred lines employed for the GWAS study. Contrarily, the sources of resistance of the extra-early yellow inbred lines used for the development of the extra-early yellow F_2:3_ mapping population were different from those used for the development of the extra-early white mapping population [40].

The use of the QTLs identified for grain yield, ears per plant and *Striga* damage at 10 WAP in MAS would fast-track the development of maize inbreds and hybrids with high grain yield for the *Striga* endemic zones of SSA. No QTL was identified for number of emerged *Striga* plants in the present study. However, the dependence on *Striga* tolerance resulting from the presence of QTL for *Striga* damage without the QTL for number of emerged *Striga* plants would allow the existing *Striga* seeds in the soil to germinate, become attached to the maize plants, emerge from the soil, flower and produce more *Striga* seeds each season. This would lead to increased *Striga* seed bank in the soil resulting in increased *Striga* infestation. In a recent study by Badu-Apraku et al [39] one QTL explaining 3.2% PVE was identified for number of emerged *Striga* plants on chromosome 3, across *Striga*-infested environments. Fortunately, even though no QTL was identified for the number of emerged *Striga* plants in the present study, early maturing maize inbred lines with positive and significant GCA effects for grain yield and significant negative GCA effects for number of emerged *Striga* plants as well as *Striga* damage have been detected in the IITA-MIP and are being used in the development of another mapping population for the identification of QTLs for number of emerged *Striga* plants. It is anticipated that the identification and use of QTL for number of emerged *Striga* plants in addition to the QTL identified in the present study would lead to increased potential effectiveness of MAS in the transfer of *Striga* resistance/tolerance genes into *Striga* susceptible maize genotypes. This would prevent the increase in the *Striga* seed bank in the soil and thus accelerate the genetic gains from selection for *Striga* resistance/tolerance and grain yield and lead to enhanced maize production and productivity in *Striga* endemic zones of SSA.

The results of the QTL analysis across individual environments revealed *qgy-3* as the largest effect QTL followed by *qgy-7*.*1* and *qepp-6*. The identified QTL explained a reliable expression of phenotypic variation varying from 3.0 to 18.5%. The QTL *qgy-3* with the largest effect was identified for *Striga* damage under *Striga* infestation on chromosome 3 with peak at 168.1cM. In the present study, detected QTL showed varying levels of additive and dominance effects for tolerance to *Striga*, indicating the relevance of both modes of gene action in controlling the inheritance of the trait. Positive and/or negative additive effects of QTL identified for grain yield, *Striga* damage and number of ears per plant suggested that favourable alleles from the *Striga* resistant/tolerant inbred, TZEEI 29 and *Striga* susceptible inbred, TZEEI 23 on the different chromosomes could be used to increase grain yield, number of ears per plant under *Striga* infestation as well as improve *Striga* tolerance through reduced *Striga* damage.

QTLs identified in the present study were used for the identification of potential candidate genes (Table 5). The QTL *qsd-3* detected for *Striga* damage was associated with the gene model GRMZM2G079290 which encodes the psk2 (phytosulfokine2) gene. The *psk2* genes have been reported to be expressed specifically in the quiescent centers of the root apical meristem where it promotes cell growth [63]. The candidate genes GRMZM2G180328 and GRMZM2G456568 associated with ears per plant and *Striga* damage encode the NAC transcription factor proteins. The NAC proteins are key regulators of plant developmental processes which include formation of lateral roots, development of shoot apical meristem and secondary cell wall biosynthesis [64]. Another candidate gene associated with grain yield and *Striga* damage, GRMZM2G087875, encodes the putative cytochrome P450 superfamily protein (cyp26). The cytochrome P450 (CYP) superfamily plays crucial roles in promoting plant growth and development and protecting plants from stresses through several biosynthetic and detoxification pathways which include hormone metabolism and biosynthesis of phytoalexin and some other secondary metabolites [65, 66].

The gene model GRMZM2G065971 linked to the QTL for grain yield and *Striga* damage encodes a magnesium transporter protein, mgt8. Plants acquire magnesium needed for growth and development from the environment and distribute within the plants in the ionic form via Mg^2+^- permeable transporters [67]. The Mg^2+^ transporters mediate Mg^2+^ uptake, translocation, and sequestration into cellular storage compartments [68, 69]. Furthermore, the gene model GRMZM2G085113 (*qgy-3*) associated with grain yield under *Striga* infestation encodes the terminal ear1 protein, te1. The terminal ear1 protein is involved in the regulation of leaf initiation rate and shoot development. It acts predominantly in the early stages of leaf development, rather than in the later stages [70, 71]. Five putative candidate genes linked to grain yield, *Striga* damage and ears per plant were found to be associated with the bHLH-transcription factors. The bHLH-transcription factors have been reported to regulate the jasmonic acid signal pathway and play essential roles in the regulation of plant defense and developmental processes [72–74].

Candidate genes GRMZM2G125653, GRMZM2G169149 and GRMZM2G143204 which encode the WRKY transcription factors were associated with grain yield and *Striga* damage and have been proven to play important roles in plant defense responses to attacks by several pathogens and parasitic weeds [75]. For example, Mutuku et al [76] found the WRKY transcription factor 45 as being essential in modulating resistance against *Striga hermonthica* by positively regulating both salicylic acid/benzothiadiazole and jasmonic acid pathways. Some of the identified putative candidate genes were found to be associated with AP2-EREBP and bZIP transcription factors. The AP2-EREBP transcription factors characterized by the presence of highly conserved AP2/ERF DNA binding domain have been reported to be mainly involved in jasmonic acid and ethylene signal transduction, while WRKY and bZIP transcription factors are mostly involved in salicylic acid mediated signal transduction [77]. Phytohormones such as salicylic acid, ethylene, jasmonate and abscisic acid initiate effective defense responses by activating defense gene expression in plants [78]. Similarly, Badu-Apraku et al [39] identified the major transcription factor families AP2/ERF, MYB, bHLH, WRKY as well as bZIP involved in plant defense signaling to be associated with QTLs for *Striga* resistance/tolerance.

Further studies on the putative candidate genes identified in the present study could provide more insights into their potential use in *Striga* resistance/tolerance breeding. Validation of QTL is crucial for a successful molecular breeding program. Discovery of QTL has little contribution crop improvement programs unless validated across environments and in different populations [79, 80]. Therefore, the QTLs expressed across *Striga* infested environments in the present study are mainly candidates for subsequent validation and gene introgression into other maize genotypes to verify their reproducibility in different environments and genetic backgrounds. In subsequent studies, fine mapping of QTLs identified in the present study would be performed to promote isolation of putative *Striga* resistance/tolerance genes by directly examining sequences in the QTL confidence intervals.

## Conclusions

In the present study, a BC_1_S_1_ mapping population derived from the *Striga* resistant/tolerant inbred line, TZEEI 29 and the *Striga* susceptible inbred line TZEEI 23 were used to understand the genetic architecture of *Striga* resistance/tolerance in tropical extra-early maturing maize genotypes. A total of 14 QTLs linked to three *Striga* resistant/tolerant traits across the two environments were detected with the proportion of phenotypic variance explained ranging from 3.0 to 18.5%. Putative candidate genes which encode major transcription factor families WRKY, bHLH, AP2-EREBPs, MYB, and bZIP involved in plant defense signaling were detected for *Striga* resistance/tolerance indicator traits. Extra-early-white mapping populations of different genetic backgrounds are presently being developed at IITA for the validation of identified QTL so that rapid introgression of *Striga* resistance genes into *Striga* susceptible but outstanding maize genotypes using MAS approaches could be a reality in SSA.

## Supporting information

S1 Fig Pedigree verification analysis of 260 BC_1_F_1_ families using SNP marker data. (TIF)

S2 Fig Linkage map of the BC_1_S_1_ mapping population derived from TZEEI 29 x TZEEI 23. Numbers on the left of each group are the map distances (cM) and marker names with physical distances (bp) are on the right. Fourteen QTLs identified for grain yield, *Striga* damage and number of ears per plant are displayed in red colour.
(TIF)

S3 Fig LOD profiles for each trait. (A) Grain yield (B) Ears per plant and (C) *Striga* damage.
(TIF)

S1 Table Results of quality control analysis of F_1_ individuals. (CSV)

S2 Table Summary statistics of the pedigree verification analysis of 260 BC_1_F_1_ families using SNP marker data. (XLSX)

S3 Table Candidate genes associated with the identified QTL for key *Striga* resistance/tolerance indicator traits under artificial *Striga* infestation. (XLSX)

S4 Table Genotypic data of the 198 BC_1_S_1_ individuals. (CSV)

S5 Table Least square means of *Striga* resistance indicator traits of 198 BC_1_S_1_ mapping population plus the two parental inbred lines evaluated across two test environments. (CSV)

## References

[1] ten Berge HFM, Hijbeek R, van Loon MP, Rurinda J, Tesfaye K, Zingore S, et al Maize crop nutrient input requirements for food security in sub-Saharan Africa. Glob Food Sec. 2019; 23: 9–21.

[2] Tesfaye K, Gbegbelegbe S, Cairns EJ, Shiferaw B, Prasanna BM, Sonder K, et al Maize systems under climate change in sub-Saharan Africa: potential impacts on production and food security. Int J of Clim Chang Str. 2015;7(3): 247–271.

[3] Badu-Apraku B, Talabi AO, Fakorede MAB, Fasanmade Y, Gedil M, Magorokosho C, et al Yield gains and associated changes in an early yellow bi-parental maize population following genomic selection for *Striga* resistance and drought tolerance. BMC Plant Biol. 2019;19: 129. 30953477 10.1186/s12870-019-1740-z PMC6451270

[4] Gurney AL, Slate J, Press MC, Scholes JD. A novel form of resistance in rice to the angiosperm parasite *Striga hermonthica*. New Phytol. 2006;169: 199–208. 16390431 10.1111/j.1469-8137.2005.01560.x

[5] Kountche BA, Jamil M, Yonli D, Nikiema MP, Blanci-Ania D, Asami T et al Suicidal germination as a control strategy for *Striga hermonthica* (Benth.) in small holder farms of sub-Saharan Africa. Plants, People, Planet 2019;1: 107–118.

[6] Lagoke STO, Parkinson V, Agunbiade RM. Parasitic weeds and control methods in Africa. In Combating *Striga* in Africa: Proceedings of the international workshop organized by IITA, ICRISAT and IDRC, 22–24 Aug 1988, IITA, Ibadan, Nigeria. ed. S.K. Kim, 1991: 3–14.

[7] Oswald A. *Striga* control-technologies and their dissemination. Crop Prot. 2005:24(4) 333–342.

[8] Frost DL, Gurney AL, Press MC, Scholes JD. *Striga hermonthica* reduces photosynthesis in sorghum: The importance of stomatal limitations and a potential role for ABA? Plant, Cell Environ. 1997;20: 4873–4492.

[9] DeVries J. The inheritance of *Striga* reactions in maize. In Breeding for *Striga* resistance in cereals, Proceeding of Workshop. IITA, Ibadan, ed. BIG. Haussmann et al., Weikersheim: Margraf Verlag. 2000: 73–84.

[10] Kim SK. Breeding maize for *Striga* tolerance and the development of a field infestation technique. In Combatting *Striga* in Africa, Proceeding of Intl. Workshop. IITA, ICRISAT, and IDRC, 22–24 Aug 1988, ed. S.K. Kim, Ibadan, Nigeria: IITA. 1991. pp. 96–108.

[11] Kim SK. Genetics of maize tolerance of *Striga hermonthica*. Crop Sci. 1994;34: 900–907.

[12] Haussmann BI, Hess DE, Welz HG, Geiger HH. Improved methodologies for breeding *Striga* resistant sorghums. Field Crops Res. 2000;66: 185–211.

[13] Rodenburg J, Diagne A, Oikeh S, Futakuchi K, Kormawa PM, Semon M et al Achievements and impact of NERICA on sustainable rice production in sub-Saharan Africa. Int Rice Comm Newsl. 2006;55: 45–58.

[14] Badu-Apraku B, Akinwale RO. Cultivar evaluation and trait analysis of tropical early maturing maize under *Striga*-infested and *Striga*-free environments. Field Crops Res. 2011;121: 186–194.

[15] Ejeta G, Butler LG, Babiker AG. New approaches to the control of *Striga* In *Striga* Research at Purdue University. Research Bulletin RB-991. Agricultural Experiment Station, Purdue University, West Lafayette, IN 1991.

[16] Rodenburg J, Bastiaans L, Weltzien E, Hess DE. How can field selection for *Striga* resistance and tolerance in sorghum be improved? Field Crops Res. 2005;93: 34–50.

[17] Pierce S, Mbwa AM, Press MC, Scholes JD. Xenognosin production and tolerance to *Striga asiatica* infection of high-yielding maize cultivars. Weed Res. 2003;43: 139–145.

[18] Rodenburg J, Bastiaans L. Host‐plant defence against *Striga* spp.: reconsidering the role of tolerance. Weed Res. 2011;51: 438–441.

[19] Berner DK, Kling JG, Singh BB. *Striga* research and control: a perspective from Africa. Plant Dis. 1995;97: 652–660.

[20] Akanvou L, Doku EV, Kling J. Estimates of genetic variances and interrelationships of traits associated with *Striga* resistance in maize. Afr Crop Sci. J. 1997;5: 1–8.

[21] Badu-Apraku B, Menkir A, Lum AF. Genetic variability for grain yield and components in an early tropical yellow maize population under *Striga hermonthica* infestation. J Crop Improv. 2007;20: 107–122.

[22] Badu-Apraku B. Genetic variances and correlations in an early tropical white maize population after three cycles of recurrent selection for *Striga* resistance. Maydica. 2007;52: 205–217.

[23] Yallou CG, Menkir A, Adetimirin VO, Kling JG. Combining ability of maize inbred lines containing genes from *Zea diploperennis* for resistance to *Striga hermonthica* (Del.) Benth. Plant Breed. 2009;128: 143–148.

[24] Kling JG, Fajemisin JM, Badu-Apraku B, Diallo A, Menkir A, Melake-Berhan A. *Striga* resistance breeding in maize. In Breeding for *Striga* resistance in cereals, ed. B.I.G. Haussmann et al., 103–118. Proceedings of a Workshop held at IITA, Ibadan, Nigeria, 16–20 Aug 1999, Margraf Verlag, Weikersheim.

[25] Gethi JG, Smith ME. Genetic responses of single crosses of maize to *Striga hermonthica* (Del.) Benth. and *Striga asiatica* (L.) Kuntze. Crop Sci. 2004;44: 2068–2077.

[26] Ramekar RV, Sa KJ, Park KC, Roy N, Kim NS, Lee JK. Construction of genetic linkage map and identification of QTLs related to agronomic traits in maize using DNA transposon-based markers. Breed Sci. 2018; 68(4): 465–473. 30369821 10.1270/jsbbs.18017 PMC6198908

[27] Maschietto V, Colombi C, Pirona R, Pea G, Strozzi F, Marocco A, et al QTL mapping and candidate genes for resistance to Fusarium ear rot and fumonisin contamination in maize. BMC Plant Biol. 2017; 17:20. 28109190 10.1186/s12870-017-0970-1 PMC5251214

[28] Wang B, Zhu Y, Zhu J, Liu Z, Liu H, Dong X, et al Identification and fine-mapping of a major maize leaf width QTL in a re-sequenced large recombinant inbred lines population. Front Plant Sci. 2018;9: 101. 29487604 10.3389/fpls.2018.00101 PMC5816676

[29] Nikolic A, Andelkovic V, Dodig D, Drinic SM, Kravic N, Micic DI. Identification of QTLs for drought tolerance in maize, II: Yield and yield components. Genetika 2013;45 (2): 341–350.

[30] Li C, Sun B, Li Y, Liu C, Wu X, Zhang D, et al Numerous genetic loci identified for drought tolerance in the maize nested association mapping populations. BMC Genomics. 2016;17: 894. 27825295 10.1186/s12864-016-3170-8 PMC5101730

[31] Trachsel S, Sun D, SanVicente FM, Zheng H, Atlin GN, Suarez EA, et al Identification of QTL for early vigor and stay-green conferring tolerance to drought in two connected advanced backcross populations in tropical maize (*Zea mays* L.). PLoS ONE 2016;11(3): e0149636. 26999525 10.1371/journal.pone.0149636 PMC4801329

[32] Silva IT. Identification of QTL associated with nitrogen metabolism in a maize (*Zea mays* L. ssp mays) testcross population”. Graduate Theses and Dissertations. 2015. Available from: https://lib.dr.iastate.edu/etd/14888/.

[33] Ribeiro PF, Badu-Apraku B, Gracen VE, Danquah EY, Garcia-Oliveira AL, Asante MD et al Identification of Quantitative Trait Loci for grain yield and other traits in tropical maize under high and low soil-nitrogen environments. Crop Sci. 2018;58: 321–331.

[34] Gowda M, Das B, Makumbi D, Babu R, Semagn K, Mahuku G, et al Genome-wide association and genomic prediction of resistance to maize lethal necrosis disease in tropical maize germplasm. Theor Appl Genet 2015;128(10): 1957–1968. 26152570 10.1007/s00122-015-2559-0 PMC4572053

[35] Wanlayaporn K, Authrapun J, Vanavichit A, Tragoonrung S. QTL Mapping for partial resistance to southern corn rust using RILs of tropical sweet corn. Am J Plant Sci. 2013; 4: 878–889.

[36] Wang AY, Li Y, Zhang CQ. QTL mapping for stay-green in maize (*Zea mays*). Can J Plant Sci. 2012;92: 249–256.

[37] Amusan IO. Mechanisms and quantitative trait loci for *Striga hermonthica* resistance in maize (*Zea mays* L.) inbred line. PhD. thesis, Purdue University. 2010. Available from: https://docs.lib.purdue.edu/dissertations/AAI3413768/.

[38] Adewale SA, Badu-Apraku B, Akinwale RO, Paterne AA, Gedil M, Garcia-Oliveira A.L. Genome-wide association study of *Striga* resistance in early maturing white tropical maize inbred lines. BMC Plant Biol. 2020, 20, 203. 32393176 10.1186/s12870-020-02360-0 PMC7212567

[39] Badu-Apraku B, Adewale S, Paterne A, Gedil M, Asiedu R. Identification of QTLs controlling resistance/tolerance to *Striga hermonthica* in an extra-early maturing yellow maize population. Agronomy 2020;10: 1168.

[40] Badu-Apraku B, Fakorede MAB. Advances in genetic enhancement of early and extra-early maize for sub-Saharan Africa. Springer; 2017.

[41] Gowda M, Worku M, Nair SK, Palacios-Rojas N, Huestis G, Prasanna BM. Quality Assurance/ Quality Control (QA/QC) in Maize Breeding and Seed Production: Theory and Practice. CIMMYT: Nairobi; 2017 pp. 13.

[42] Milne I, Shaw P, Stephen G, Bayer M, Cardle L, Thomas WTB et al Flapjack-graphical genotype visualization. Bioinform. (Oxford, England). 2010;26 (24): 3133–3134. 10.1093/bioinformatics/btq580 PMC2995120 20956241

[43] Soil Survey Staff. Soil taxonomy: A basic system of soil classification for making and interpreting soil surveys 2nd ed USDA–NRCS Agriculture Handbook No. 436. U.S. Gov. Print. Office, Washington, DC 1999 pp. 869.

[44] Snedecor GW, Cochran WG. Statistical methods, 8thEdn Ames: Iowa State Univ Press Iowa 1989.

[45] SAS Institute Inc. Statistical Analysis Software (SAS) user’s guide. Cary: SAS Inst; 2011.

[46] Holland JB, Nyquist WE, Cervantes-Martiinez CT. Estimating and interpreting heritability for plant breeding: An update In: Janick J., editor, Plant breeding reviews. John Wiley & Sons, New York 2003;22: 9–111.

[47] Sansaloni CP, Petroli CD, Jaccoud D, Carling J, Detering F, Grattapaglia D, et al Diversity Arrays Technology (DArT) and next-generation sequencing combined: genome-wide, high throughput, highly informative genotyping for molecular breeding of Eucalyptus. BMC Proc. 2011;5: 54.

[48] Chen J, Zavala C, Ortega NG, Petroli CD, Franco J, Burgüeño JA, et al The development of quality control genotyping approaches: A case study using elite maize lines. PLoS ONE. 2016: 11(6):e 0157236. 10.1371/journal.pone.0157236 PMC4900658 27280295

[49] Xu S., Hu Z. Mapping quantitative trait loci using distorted markers. Int. J. Plant Genom. 2009: 10.1155/2009/410825 PMC2825659 20182628

[50] Broman KW, Wu H, Sen Ś, Churchill GAJ. Bioinform. 2003;19: 889–890. 10.1093/bioinformatics/btg112 12724300

[51] Arends D, Prins P, Jansen RC, Broman KW. R/qtl: high-throughput multiple QTL mapping. J Bioinform. 2010;26(23): 2990–2992. 10.1093/bioinformatics/btq565 PMC2982156 20966004

[52] Weng Y, Colle M, Wang Y, Yang L, Rubinstein M, Sherman A, et al QTL mapping in multiple populations and development stages reveals dynamic quantitative trait loci for fruit size in cucumbers of different market classes. Theor Appl Genet. 2015; 128:1747–63. 26048092 10.1007/s00122-015-2544-7

[53] van Ooijen JW. Accuracy of mapping quantitative trait loci in autogamous species. Theor Appl Genet. 1992;84: 803–811. 24201478 10.1007/BF00227388

[54] Lübberstedt T, Melchinger AE, Schön CC, Utz HF, Klein D. QTL mapping in testcrosses of European flint lines of maize: I. Comparison of different testers for forage yield traits. Crop Sci. 1997;37: 921–931.

[55] Bo KL, Ma Z, Chen JF, Weng Y. Molecular mapping reveals structural rearrangements and quantitative trait loci underlying traits with local adaptation in semi-wild Xishuangbana cucumber (*Cucumis sativus* L. var. xishuangbannanesis Qi et Yuan). Theor Appl Genet. 2015;128(1): 25–39. 25358412 10.1007/s00122-014-2410-z

[56] Gedil M, Menkir A. An integrated molecular and conventional breeding scheme for enhancing genetic gain in maize in Africa. Front Plant Sci. 2019;10: 1430 10.3389/fpls.2019.01430 31781144 PMC6851238

[57] Badu-Apraku B, Menkir A, Ajala SO, Akinwale RO, Oyekunle M, Obeng-Antwi K. Performance of tropical early maturing maize cultivars in multiple stress environments. Can. J Plant Sci. 2010;90: 831–852.

[58] Akinwale RO, Badu-Apraku B, Fakorede MAB. Evaluation of *Striga*-resistant early maize hybrids and test locations under *Striga*-infested and *Striga*-free environments. Afr Crop Sci J. 2013:21(1): 1–19.

[59] Han S, Yuan M, Clevenger JP, Li C, Hagan A, Zhang X, et al SNP-Based linkage map revealed QTLs for resistance to early and late leaf spot diseases in peanut (*Arachis hypogaea* L.). Front Plant Sci. 2018;9: 1012. 30042783 10.3389/fpls.2018.01012 PMC6048419

[60] Ertiro TB, Olsen M, Das B, Gowda M, Labuschagne M. Genetic dissection of grain yield and agronomic traits in maize under optimum and low-nitrogen stressed environments. Int J Mol Sci. 2020;21: 543. 10.3390/ijms21020543 PMC7013417 31952130

[61] Samayoa LF, Malvar RA, McMullen MD, Butrón A. Identification of QTL for resistance to Mediterranean corn borer in a maize tropical line to improve temperate germplasm. BMC Plant Biol. 2015;15: 265. 26530038 10.1186/s12870-015-0652-9 PMC4632334

[62] Zhao X, Peng Y, Zhang J, Fang P, Wu B. Identification of QTLs and Meta-QTLs for seven agronomic traits in multiple maize populations under well-watered and water-stressed conditions. Crop Sci. 2018;58: 507–520.

[63] Sauter M. Phytosulfokine peptide signaling J Exp Bot. 2015;66 (17): 5161–5169. 10.1093/jxb/erv071 25754406

[64] Liu X, Wang T, Bartholomew E, Black K, Dong M, Zhang Y, et al Comprehensive analysis of NAC transcription factors and their expression during fruit spine development in cucumber (*Cucumis sativus* L.). Hortic. Res. 2018; 5:31. 29872536 10.1038/s41438-018-0036-z PMC5981648

[65] Jun X, Xin-yu W, Wang-zhen G. The cytochrome P450 superfamily: Key players in plant development and defense. J. Integr. Agric 2015;14(9): 1673–1686.

[66] Pandian BA, Sathishraj R, Djanaguiraman M, Prasad PV, Jugulam M. Role of cytochrome P450 enzymes in plant stress response. Antioxidants 2020;9: 454. 10.3390/antiox9050454 PMC7278705 32466087

[67] Yan Y-W, Mao D-D, Yang L, Qi J-L, Zhang X-X, Tang Q-L. Magnesium transporter MGT6 plays an essential role in maintaining magnesium homeostasis and regulating high magnesium tolerance in Arabidopsis. Front. Plant Sci. 9:274. 29593754 10.3389/fpls.2018.00274 PMC5857585

[68] Hermans C, Conn SJ, Chen J, Xiao Q, Verbrugeen N. An update on magnesium homeostasis mechanisms in plants. Biometals 2013;5: 1170–1183. 10.1039/c3mt20223b 23420558

[69] Li H, Du H, Huang K, Chen X, Liu T, Gao. Identification, and functional and expression analyses of the CorA/MRS2/MGT-Type Magnesium transporter family in maize. Plant Cell Physiol. 2016;57(6): 1153–1168. 27084594 10.1093/pcp/pcw064

[70] Veit B, Briggs SP, Schmidt RJ, Yanofsky MF, Hake S. Regulation of leaf initiation by the terminal ear 1 gene of maize. Nature, 1998;393: 166–168. 9603518 10.1038/30239

[71] Zhang D, Sun W, Singh R, Zheng Y, Cao Z, Li M. et al GRF-interacting factor1 (gif1) regulates shoot architecture and meristem determinacy in maize. Plant Cell Advance Publication. 2018; 10.1105/tpc.17.00791 PMC5868708 29437990

[72] Song S, Qi T, Fan M, Zhang X, Gao H, Huang H. et al The bHLH subgroup iiid factors negatively regulate jasmonate-mediated plant defense and development. PLoS Genet 2013;9(7): e1003653. 23935516 10.1371/journal.pgen.1003653 PMC3723532

[73] Zhao F, Li G, Hu P, Zhao X, Li L, Wei W. et al Identification of basic/helix-loop helix transcription factors reveals candidate genes involved in anthocyanin biosynthesis from the strawberry white-flesh mutant. Sci. Rep. 2018;8:2721. 29426907 10.1038/s41598-018-21136-z PMC5807450

[74] Meraj TA, Fu J, Raza MA, Zhu C, Shen Q, Xu D. et al Transcriptional factors regulate plant stress responses through mediating secondary metabolism. Genes 2020;11: 346. 10.3390/genes11040346 PMC7230336 32218164

[75] Jiang J, Ma S, Ye N, Jiang M, Cao J, Zhang1 J. WRKY transcription factors in plant responses to stresses. J. Integr. Plant Biol. 2017;59(2): 86–101. 27995748 10.1111/jipb.12513

[76] Mutuku JM, Yoshida S, Shimizu T, Ichihashi Y, Wakatake T, Takahashi A et al The WRKY45-dependent signaling pathway is required for resistance against *Striga hermonthica* parasitism. Plant Physiol. 2015;168: 1152–1163. 26025049 10.1104/pp.114.256404 PMC4741350

[77] Rawat N. Plant defense gene regulation and transcription factor dynamics. J Rice Res. 2016;4: e128.

[78] Li N, Han X, Feng D, Yuan D, Huang L-J. Signaling crosstalk between salicylic acid and ethylene/jasmonate in plant defense: Do we understand what they are whispering? Int. J. Mol. Sci. 2019;20: 671. 10.3390/ijms20030671 PMC6387439 30720746

[79] Gelli M, Konda AR, Liu K, Zhang Chi, Clemente TE, Holding DR, et al Validation of QTL mapping and transcriptome profiling for identification of candidate genes associated with nitrogen stress tolerance in sorghum. BMC Plant Biol. 2017; 17:123. 28697783 10.1186/s12870-017-1064-9 PMC5505042

[80] Awata LAO, Beyene Y, Gowda M, Suresh LM, Jumbo MB, Tongoona P, et al Genetic analysis of QTL for resistance to maize lethal necrosis in multiple mapping populations. Genes 2020; 11: 32. 10.3390/genes11010032 PMC7017159 31888105

