# Supplementary figures and images for: Identification of QTLs for grain yield and other traits in tropical maize under Striga infestation
Source: PLoS One. 2020 Sep 14;15(9):e0239205. doi: 10.1371/journal.pone.0239205 (PMC7489516; doi:10.1371/journal.pone.0239205)

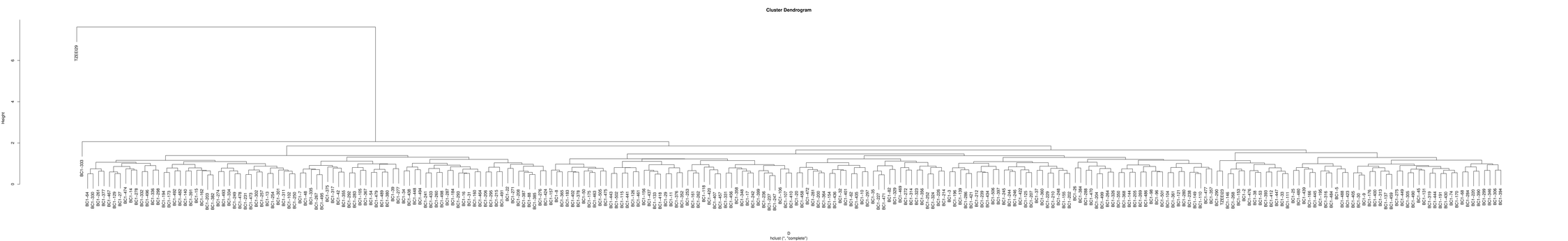

Supplement: S1 Fig — (TIF) [file pone.0239205.s001.tif]

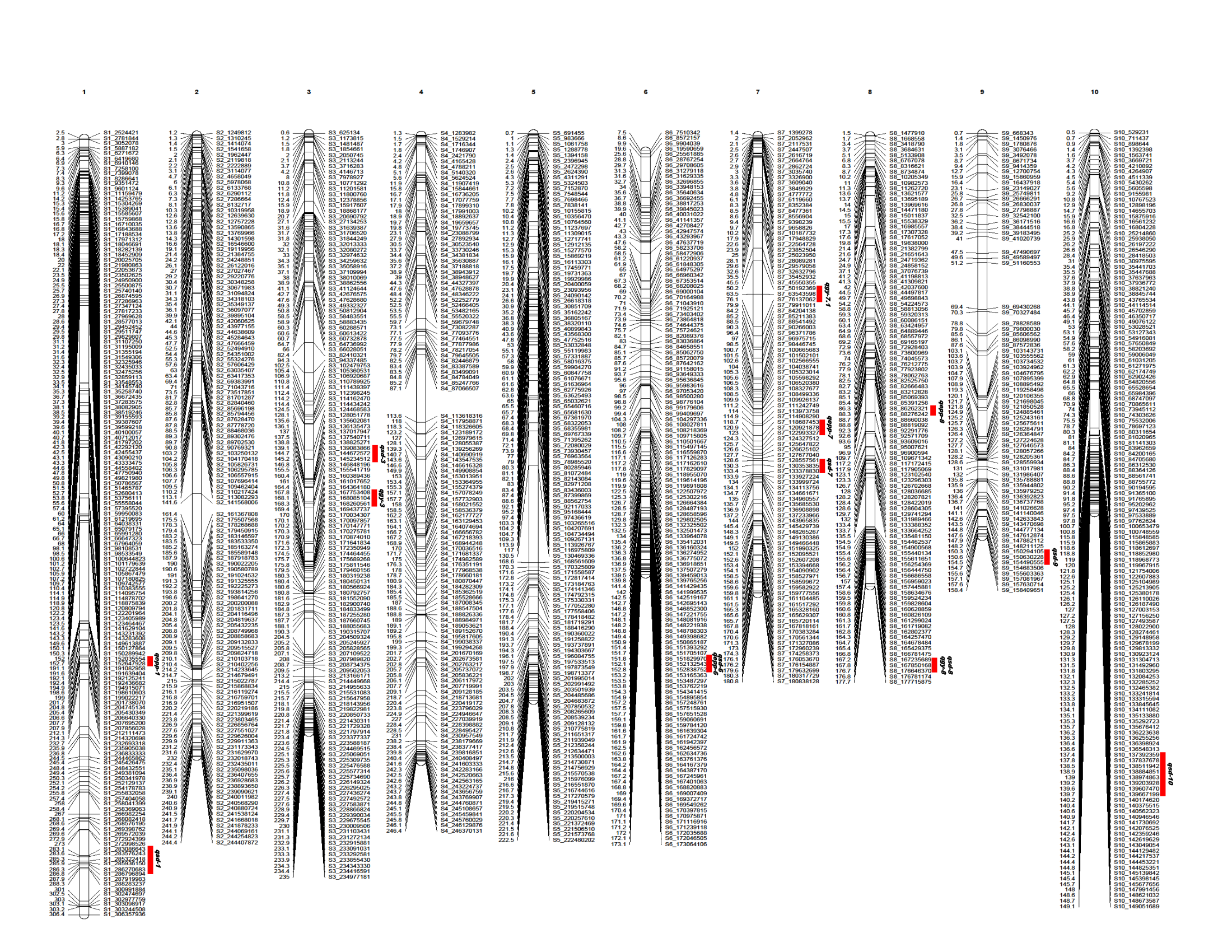

Supplement: S2 Fig — Numbers on the left of each group are the map distances (cM) and marker names with physical distances (bp) are on the right. Fourteen QTLs identified for grain yield, Striga damage and number of ears per plant are displayed in red colour. (TIF) [file pone.0239205.s002.tif]

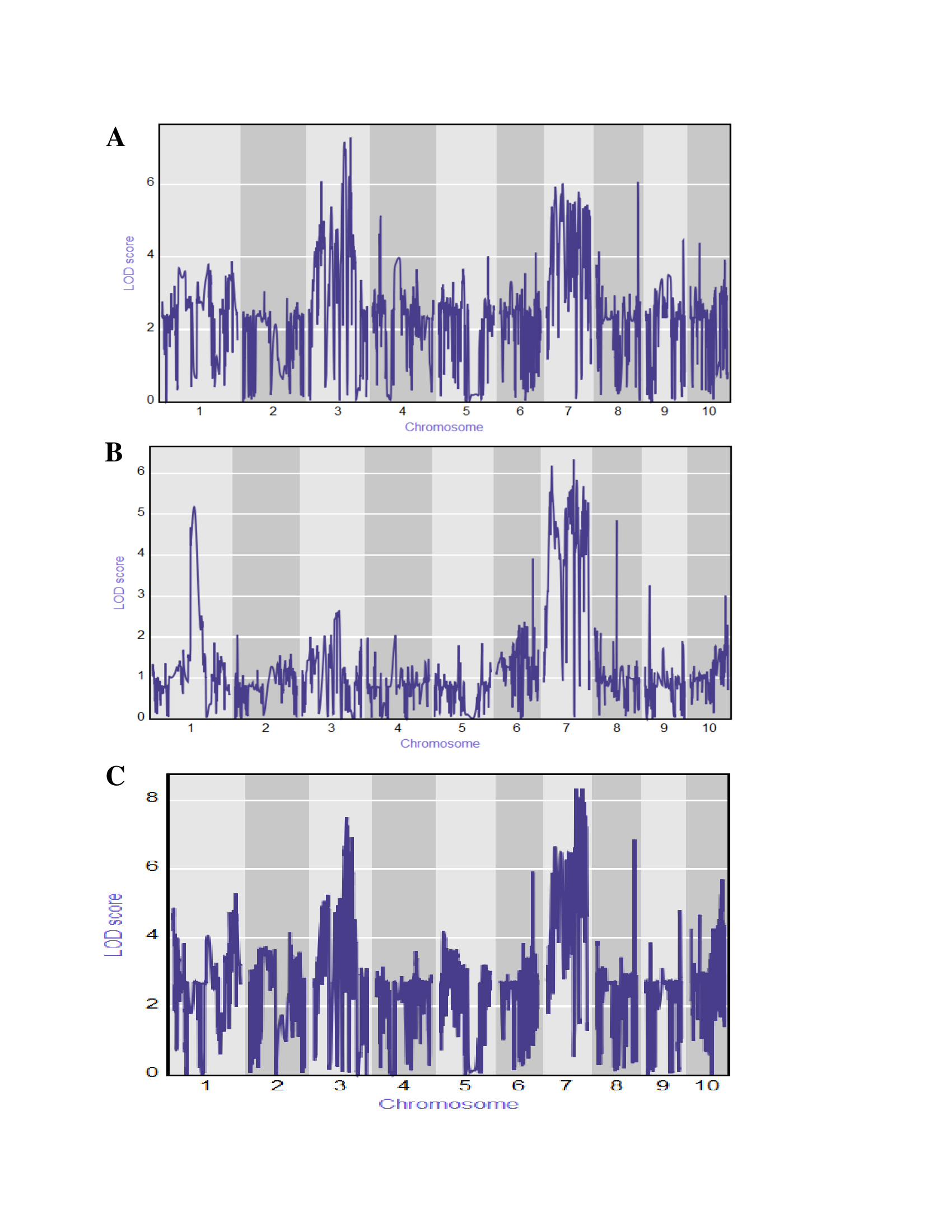

Supplement: S3 Fig — (A) Grain yield (B) Ears per plant and (C) Striga damage. (TIF) [file pone.0239205.s003.tif]
